# Auditory–limbic–cerebellum interactions and cognitive impairments in noise‐induced hearing loss

**DOI:** 10.1111/cns.14028

**Published:** 2022-11-15

**Authors:** Xiao‐Min Xu, Yuan Feng, Jian Wang, Richard Salvi, Xindao Yin, Jun Gao, Yu‐Chen Chen

**Affiliations:** ^1^ Department of Radiology, Nanjing First Hospital Nanjing Medical University Nanjing China; ^2^ School of Human Communication Disorders Dalhousie University Halifax Nova Scotia Canada; ^3^ Center for Hearing and Deafness University at Buffalo Buffalo New York USA; ^4^ The Department of Neurobiology, Key Laboratory of Human Functional Genomics of Jiangsu Nanjing Medical University Nanjing China

**Keywords:** auditory cortex, cognitive impairment, hippocampus, limbic system, noise‐induced hearing loss

## Abstract

**Aims:**

This study aimed to explore the neural substrate of hearing loss‐related central nervous system in rats and its correlation with cognition.

**Methods:**

We identified the neural mechanism for these debilitating abnormalities by inducing a bilateral hearing loss animal model using intense broadband noise (122 dB of broadband noise for 2 h) and used the Morris water maze test to characterize the behavioral changes at 6 months post‐noise exposure. Functional magnetic resonance imaging (fMRI) was conducted to clarify disrupted functional network using bilateral auditory cortex (ACx) as a seed. Structural diffusion tensor imaging (DTI) was applied to illustrate characteristics of fibers in ACx and hippocampus. Pearson correlation was computed behavioral tests and other features.

**Results:**

A deficit in spatial learning/memory, body weight, and negative correlation between them was observed. Functional connectivity revealed weakened coupling within the ACx and inferior colliculus, lateral lemniscus, the primary motor cortex, the olfactory tubercle, hippocampus, and the paraflocculus lobe of the cerebellum. The fiber number and mean length of ACx and different hippocampal subregions were also damaged in hearing loss rats.

**Conclusion:**

A new model of auditory–limbic–cerebellum interactions accounting for noise‐induced hearing loss and cognitive impairments is proposed.

## Graphical abstract

The present study investigated a new model of auditory‐limbic‐arousal‐cerebellum interactions accounting for noise induced hearing loss and cognitive impairments. Combining functional and structural MRI might provide different aspects of brain plasticity, which could serve as a potential role in non‐invasive diagnosis and therapy evaluation of NIHL in the future.

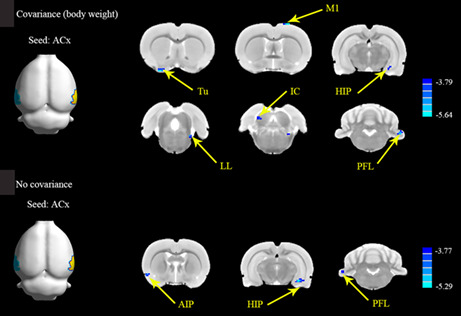

## 1 INTRODUCTION

Hearing impairment is independently associated with a 30%–40% acceleration in cognitive decline, as well as an increased rate of all‐cause dementia.^1^ According to the framework^2^ that WHO International Classification of Functioning and Disability provided, hearing loss can affect body functions and structures (deterioration in the ear), related activities (reduced speech understanding), and the participation of the individual in society (receiving education about health issues, attending social events, or receiving health services). It is reported that every 10 dB in hearing loss can lead to a 20% increased risk of developing dementia.^3^ The auditory cortex (ACx) is not only known as a major site for processing auditory information and performing high functions of hearing but also related to cognition.^4^, ^5^ Several evidence has proposed the relationship between hearing deprivation and cognitive impairment, but the causative connections remain to be confirmed.

Extreme noise exposure is a leading cause of acquired hearing loss^6^ and can be easily established in a quantity controllable manner as compared with other methods. Prolonged exposure to intense sounds can induce moderate–severe sensorineural hearing loss characterized by the death of outer hair cells, inner hair cells, spiral ganglion neurons, and the auditory nerve fibers that project to the central auditory system.^7^ Our previous study^8^ has found anxious‐ and depression‐like behaviors in the early‐stage post‐noise, but the long‐term effects of auditory deprivation in cognitive decline are poorly understood. Shortly after noise exposure, the role of oxidative stress has been the focus in existing researches. However, the independent influence of hearing loss and underlying neurological mechanisms need to be substantiated.

Since Morris water maze (MWM) test does not require hearing function, the decline in the performance suggests that hearing loss impairs the general function of the hippocampus.^9^ Moreover, there are close anatomic and functional connections between cognitive brain and the main nuclei in auditory pathway. Functional connectivity (FC) is a powerful tool to study the considerable neural plasticity in terms of whole brain. Functional connections are defined as the temporal coincidence of neurophysiological events (including anatomical structure and functional associations) and effectively describe information flow between brain areas.^10^ Additionally, diffusion tensor imaging (DTI) can reveal microstructural changes in white matter and has been widely used in animal models.^11^

In the present study, we used a broadband noise exposure for 2 h (122 dB SPL) to establish noise‐induced hearing loss (NIHL) model. Then, series of experiments (including MWM test, auditory function evaluation, and functional and structural MRI scanning) were conducted on rats in an aim to gain a more complete understanding within and beyond the auditory pathway, as well as the neural mechanism of cognitive impairments following hearing deprivation.

## 2 MATERIALS AND METHODS

### 2.1 Animal models

All animal procedures were conducted in accordance with the National Institutes of Health Guide for the Care and Use of Laboratory Animals and approved by the Animal Care Committee of Nanjing Medical University, Nanjing, China. Twenty‐six male Sprague–Dawley rats 4 weeks of age and weighing between 120 and 150 g were used in this study (Qinglongshan Animal Center, SCXK[ZHE]2014–0001). The colony room with ad libitum access to food and water was maintained at 22°C with a 12:12‐h light/dark cycle. After 1‐week acclimation, the animals were randomly assigned into the healthy controls (HCs) group (*n* = 13) and NIHL group (*n* = 13). A total of 12 control rats and 13 NIHL groups were left following 6 months. The animals were treated humanely and with regard for alleviation of suffering.

### 2.2 Experimental design

At the baseline time, we recorded the body weight and Preyer's reflex (PR) of each rat that make sure all animals have normal hearing abilities. After 1‐week acclimation, we conducted baseline MRI scan and measured the breath frequency during the same period. Then, we exposed the NIHL group to broadband intense noise (122 dB SPL) for 2 h. MWM task, auditory brainstem response (ABR) test, and MRI scanning were done 6 months later, and one normal rat was dead of anesthesia.

### 2.3 Noise exposure and auditory evaluation

Auditory brainstem response test has been used for decades to assess auditory function. This technique is non‐invasive and easy to use, making it ideal for use in both clinical and experimental studies. ABR waves are recorded far‐field from the scalp and are typically comprised of five to seven vertex positive waves that appear within the first 10 ms after an auditory stimulus.^12^ The procedures of noise exposure and ABR test were described in detail in our previous studies.^8^, ^13^

### 2.4 MWM test

The water maze used in the present study was a black circular pool with 160 cm in diameter and 60 cm in height, which was divided into four equal quadrants (I, II, III, and IV). An escape platform (11 cm in diameter) was placed in the center of quadrant III, 1–2 cm beneath the surface of water. Consistent with other researches,^14^, ^15^ this MWM test was comprised of spatial learning/training and memory test. During the learning phase, each rat entered into the maze from four different quadrants randomly for five consecutive days. On the memory test day, the platform was removed from the maze and the swimming paths in 90 s were recorded using a computer system with a video camera (AXIS‐90).

### 2.5 Functional MRI scanning and data analysis

A 7.0 T animal MRI scanner (PharmaScan, Bruker Biospin GmbH) using a quadrature surface RF coil was used to acquire functional and structural MRI data. Every rat was positioned in the scanner in a prone position with a bite bar and two rods located on the left and right sides of the temporal surface of the head. The cotton was put in both ears to attenuate noise level from MRI scanning. A temperature‐controlled water blanket was placed beneath the rat to maintain the body temperature at 37.5°C. Animals were anesthetized with 5% isoflurane for 4 min firstly, then maintained throughout scanning with 0.3% iso plus intramuscular injection of the medetomidine (0.05 mg/kg). The respiration rates were monitored by a Bruker Physiogard system with a sensor placed under the animal's chest during the whole scanning procedure.

In order to keep the blood oxygenation level‐dependent (BOLD) signal, heart beats, and respiration rates stable, MRI scanning started 1 h after the injection of anesthetic.^16^ The parameters of BOLD sequence were as follows: repetition time (TR) = 2000 ms; echo time (TE) = 18 ms; slices = 21; filed of view (FOV) = 3.2 cm × 3.2 cm; number of averages = 1; matrix = 64 × 64; slice thickness/gap = 1/0 mm; flip angle = 90°; and 100 volumes. The preprocessing steps were carried out with statistical parametric mapping software (DPARBI_2.3, http://rfmri.org/dpabi; SPM12, http://www.fil.ion.ucl.ac.uk/spm/), including: (1) excluding first 10 times to allow for scanner calibration and adaptation to the environment; (2) skull stripping; (3) slice timing; (4) realignment and correction for head motion; (5) spatial normalization to the standard rat brain stereotaxic coordinates; (6) detrending and filtering (0.01–0.1 Hz); and (7) smoothing with a full‐width at half‐maximum of 1 mm. Data with head movements exceeded 0.1 mm of maximum translation in the x, y, or z directions or 1.0 of maximum rotation about the three axes were discarded. Subsequently, we chose the bilateral auditory cortex (ACx) as a seed to explore its functional connections with other auditory and non‐auditory brain regions. Two‐sample *t‐*test was performed to identify significant changes in FC between NIHL and HCs groups and the thresholds were set at an uncorrected *p*‐value of *p* < 0.001 and cluster size >10 voxels.

### 2.6 Data Acquisition and Imaging (DTI) analysis

The DTI sequence was used to acquire 30 distinct diffusion directions and 5 reference images with the following parameters: *b*‐value = 1000 s/mm^2^; TR = 2000 ms; TE = 23 ms; FOV = 3.2 cm × 3.2 cm; matrix = 128 × 128; slice thickness = 1 mm; and 20 slices. Fiber tracking was computed using the TrackVis and Diffusion Toolkit software (http://www.trackvis.org). Regions of interest (ROI) drawing were obtained on direction‐encoded color maps with sizes of 3 mm^2^ in bilateral ACx and subregions of the hippocampus (CA1 and CA2). The number of fibers and mean fiber length in ROIs were evaluated.

### 2.7 Statistical analysis

Group differences in physiology measures and body weight were analyzed using the Student's *t*‐test. Additionally, to investigate the relationship between behavioral tests and body weight, the correlation analysis was conducted using the Pearson correlation analysis by SPSS software (version18.0; SPSS). A *p* < 0.05 was considered statistically significant and Bonferroni correction for multiple comparisons was applied in this analysis.

## 3 RESULTS

### 3.1 Basic characteristics of rats during the experiments

The basic characteristics were detailed in Table 1. One rat was excluded because of death which was induced by intolerable anesthesia. At baseline time, two groups were well matched for number, age, gender, head motion, body weight, and frequency of breath, and all rats had normal hearing. After noise exposure for 6 months, body weight showed a significant difference (*p* < 0.002). Unexpectedly, HCs group was heavier than the NIHL group, which meant the growth rate of weight was faster. It was reported that fMRI data were not limited by instrumental noise, but by fluctuations of biological origin, especially heartbeat and breathing.^17^ During our scanning, the frequency of breath between groups showed no significance.

**TABLE 1:** Basic information on rats used in each of the assessments.

Measurements	NIHL group	HCs group	*p*‐value
Baseline			
Number of rats (*n*)	13	13	/
Body weight (g)	135 ± 11	136 ± 9	0.852
Frequency of breath during MRI scanning (n/min)	66 ± 11	65 ± 11	0.690
6 months post‐noise exposure			
Number of rats (*n*)	13	12	/
Body weight (g)	457 ± 42	508 ± 31	0.002*
Frequency of breath during MRI scanning (n/min)	55 ± 3	53 ± 3	0.272

Abbreviations: HCs, healthy controls; NIHL, noise‐induced hearing loss.

* *p* < 0.01.

### 3.2 Bilateral hearing loss induced by intense noise exposure

**FIGURE 1:**
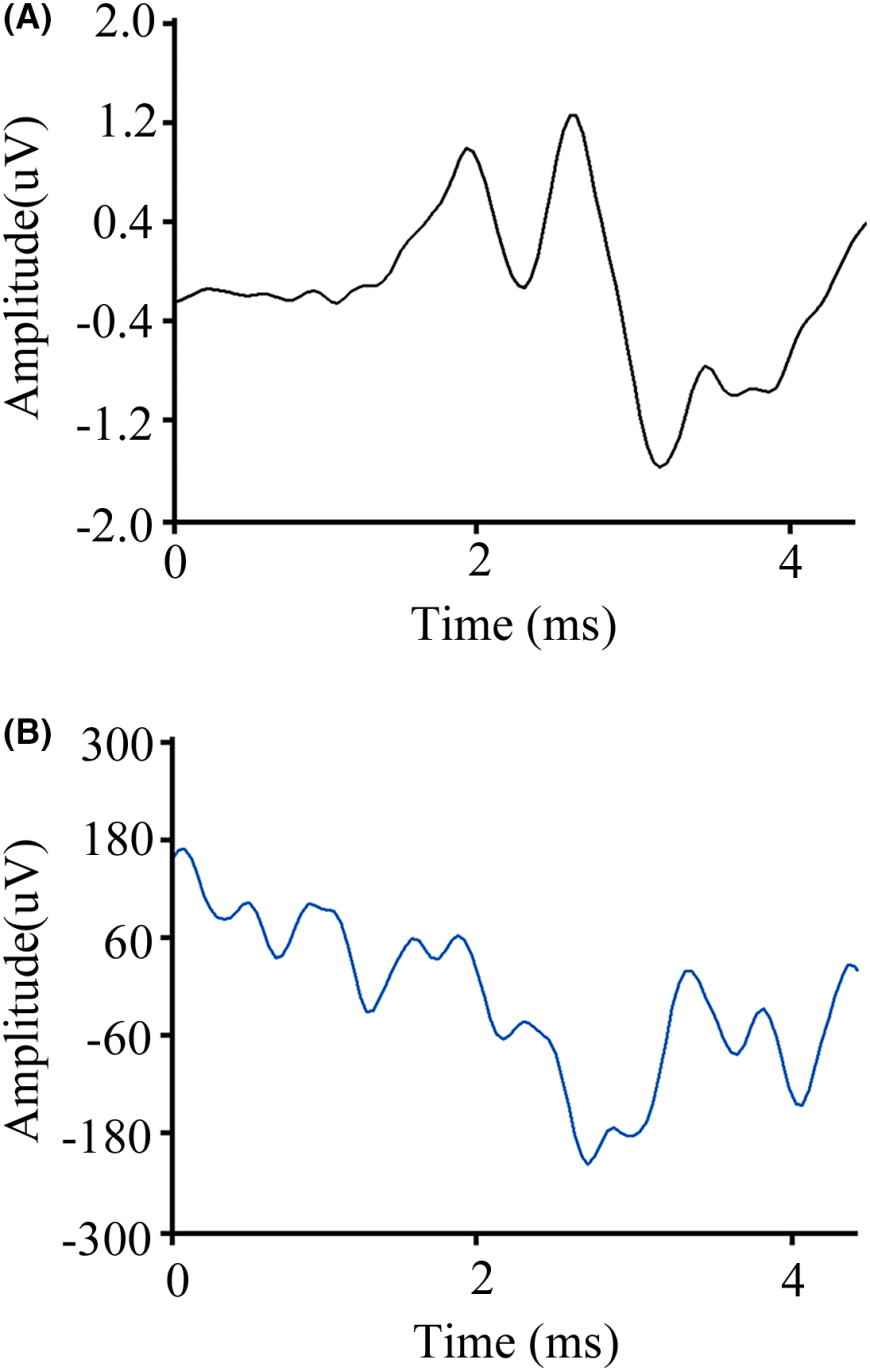
Typical ABR waveforms in the NIHL group and HCs group at 6 months post‐noise exposure. (A) Representative ABR waveforms elicited by 8 kHz tone bursts presented at 90 dB SPL in the HCs group. (B) Representative ABR waveforms elicited by 8 kHz tone bursts presented at 90 dB SPL in the NIHL group. Note prominent, well‐defined peaks in the HCs group and barely detectable response in the NIHL group. ABR, auditory brainstem response; HCs, healthy controls; NIHL, noise‐ induced hearing loss.

A brief exposure to broadband noise at 122 dB SPL produced a significant permanent threshold shift of moderate–severe degree as tested in the audiogram of ABR at 6 months post‐noise (6MPN) (Table S1). Figure 1A,B represented the typical ABR waveforms of HCs and NIHL rats, respectively. Compared with the HCs group, rats post‐noise cannot lead waveform under different stimulus frequencies, even 90 dB SPL.

### 3.3 Group differences in hippocampal memory in rats

**FIGURE 2:**
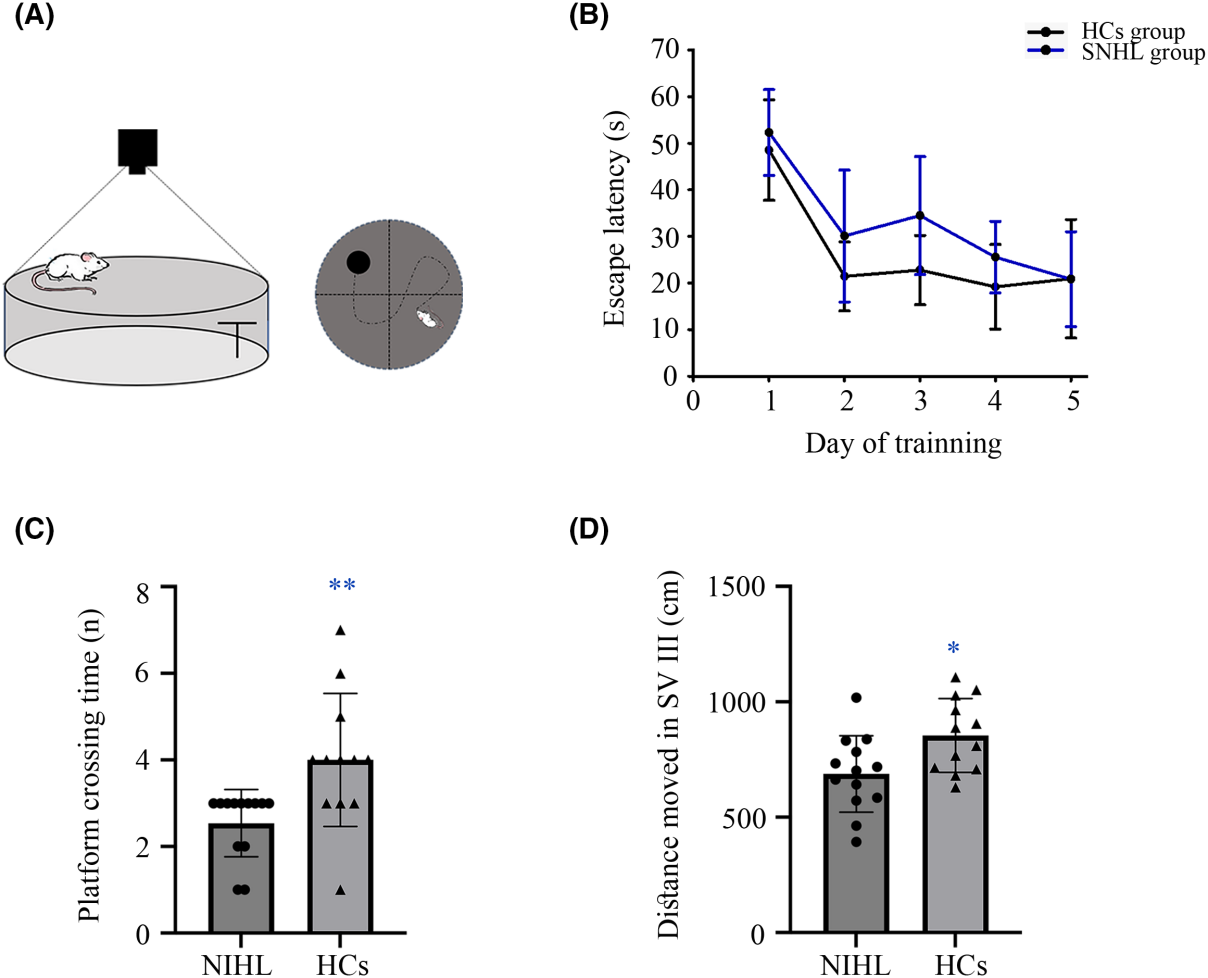
Weakened performance underlying NIHL in MWM test. (A) Schematic diagram of MWM. (B) Escape latency of NIHL and HCs group during training days. (C) Platform crossing time on the test day. (D) Distance moved in SV III on the test day. *p < 0.05; **p < 0.001. HCs, healthy controls; MWM, Morris water maze; NIHL, noise‐ induced hearing loss.

Spatial learning ability and memory were tested in an MWM (Figure 2A). Figure 2B showed the escape latency in the training session and no significance was observed. It was found that the platform crossing time of NIHL rats was less than HCs group in the spatial orientation test period (*p* = 0.006, Figure 2C). In addition, rats in the NIHL group moved more than the HCs group in the target quadrant (SV III) on the test day (*p* = 0.018, Figure 2D).

### 3.4 The alterations of functional connectivity present in rat brain

**FIGURE 3:**
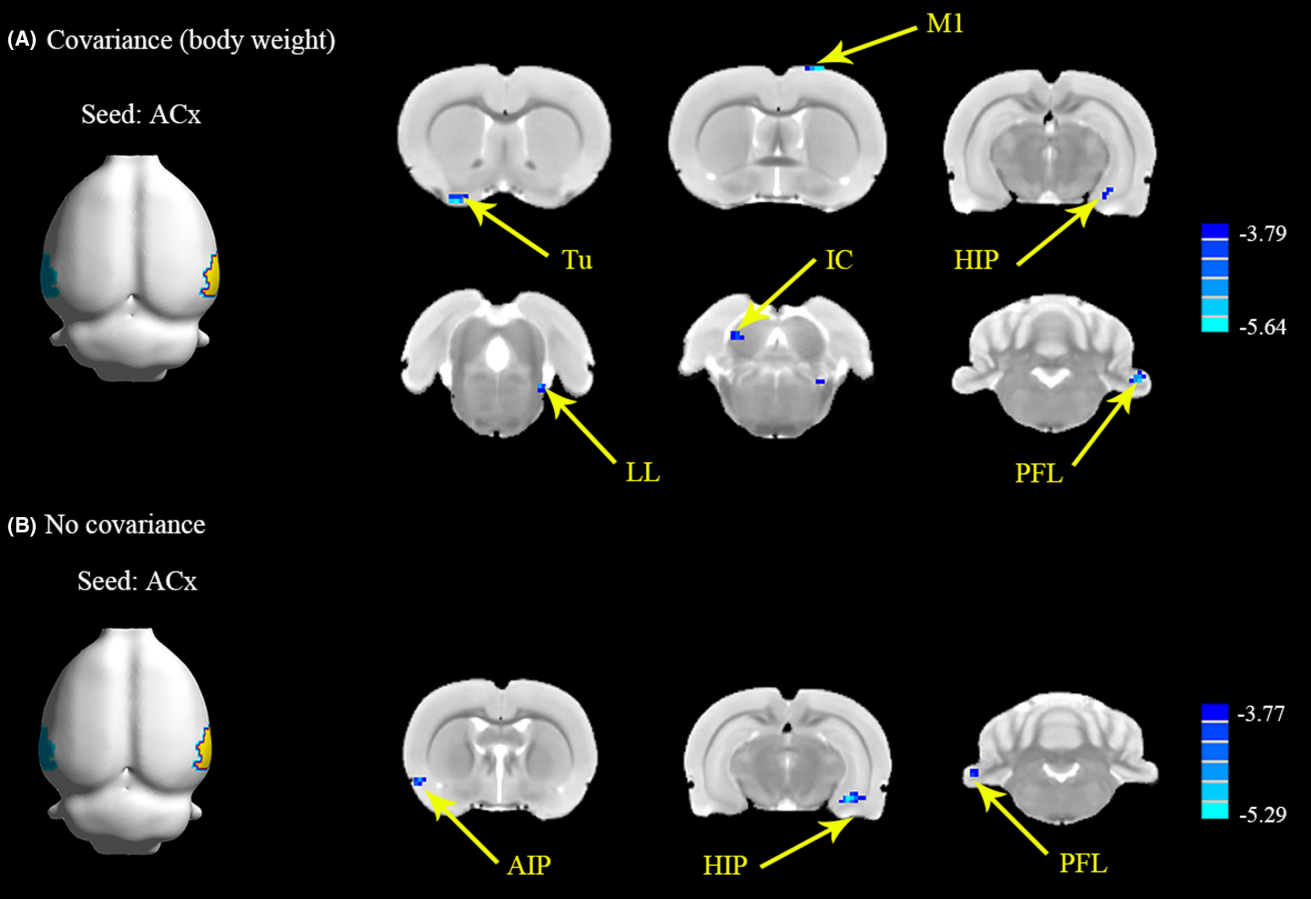
NIHL alters functional connectivity in specific brain regions using ACx as the seed. (A) The ACx shows weakened connections with Tu, M1, HIP, LL, IC, and PFL using body weight as the covariance. Scale bar is shown in lower right; *t*‐values ranged from −5.64 to −3.79. (B) The ACx shows decreased connections with AIP, HIP, and PFL using no covariance. Scale bar is shown in lower right; *t*‐values ranged from −5.29 to −3.77. ACx, primary auditory cortex; AIP, agranular insular cortex; HIP, hippocampus; IC, inferior colliculus; LL, lateral lemniscus; M1, primary motor cortex; NIHL, noise‐ induced hearing loss; PFL, parafloccular lobe of cerebellum; Tu, olfactory tubercle. (*p* < 0.001, uncorrected).

The FC pattern is shown in Figure 3 and Table S2 (cluster size, and *t*‐values shown for the left and right hemispheres). Using the bilateral ACx as a seed, the NIHL group showed a significant bilateral decrease in FC in clusters located in the paraflocculus lobe of the cerebellum (PFL, 36 voxels), inferior colliculus (IC, 11 voxels), lateral lemniscus (LL, 14 voxels), hippocampus (17 voxels), the primary motor cortex (M1, 12 voxels), and olfactory tubercle (Tu, 24 voxels), taking body weight as a covariance (Figure 3A). Meanwhile, the two‐sample *t*‐test reflected that ACx showed weaker connectivity with PFL (10 voxels), agranular insular cortex (AIP, 10 voxels), and hippocampus (30 voxels) in the NIHL group without the covariance (Figure 3B). No increases in FC were observed. Additionally, we tried to do a correlation analysis between FC and behavioral measures, but no significant results occurred.

### 3.5 Characteristics of fibers in auditory cortex and hippocampus

**FIGURE 4:**
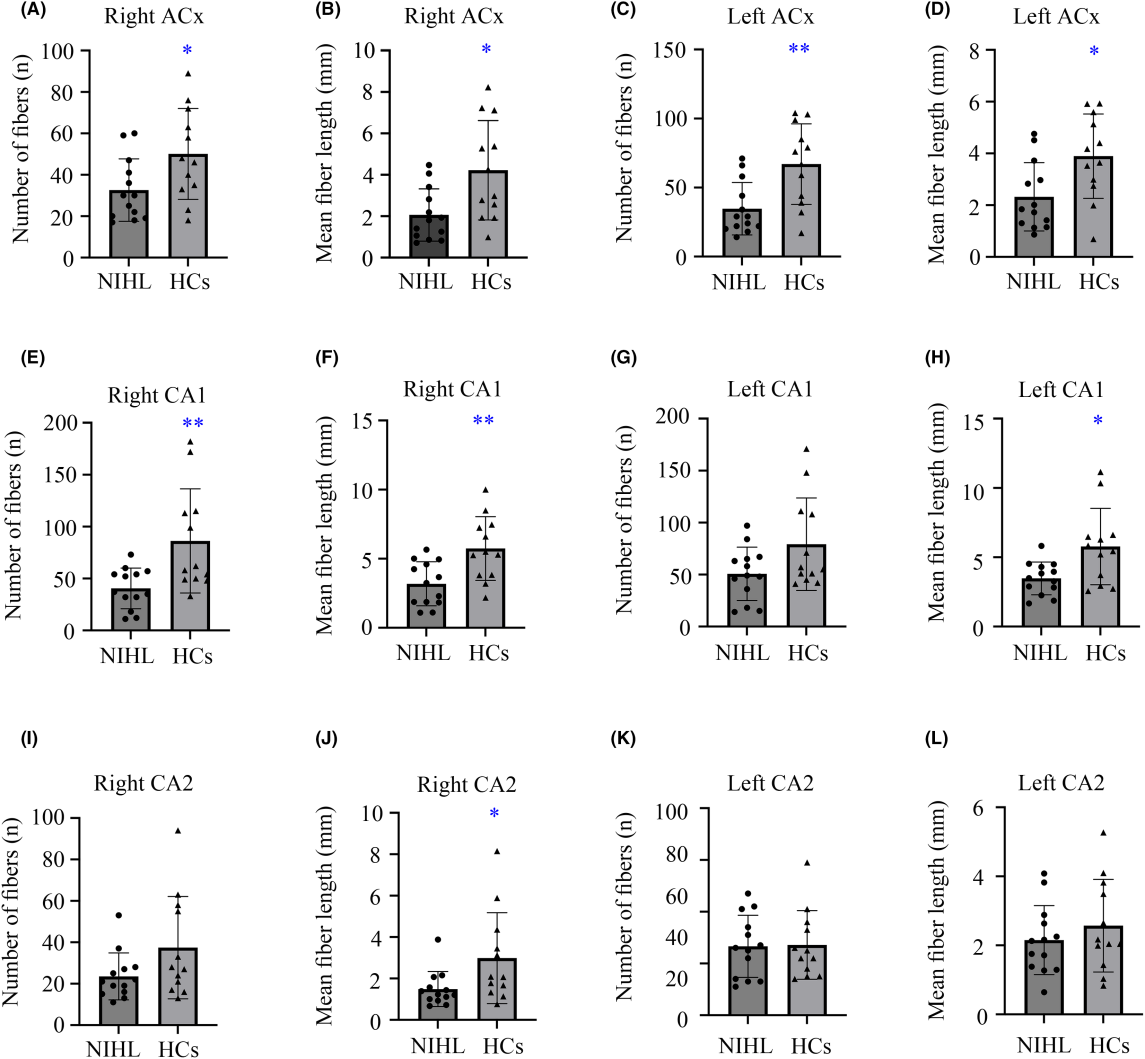
Track alterations of bilateral ACx and hippocampal subregions underlying NIHL assessed by DTI technique. (A) Number of fibers of right ACx; (B) Mean fiber length of right ACx; (C) Number of fibers of left ACx; (D) Mean fiber length of left ACx; (E) Number of fibers of right CA1; (F) Mean fiber length of right CA1; (G) Number of fibers of left CA1; (H) Mean fiber length of left CA1; (I) Number of fibers of right CA2; (J) Mean fiber length of right CA2; (K) Number of fibers of left CA2; (L) Mean fiber length of left CA2. Data are shown as mean ± SEM. ACx, primary auditory cortex; DTI, diffusion tensor imaging; NIHL, noise‐induced hearing loss.

At 6 months post‐noise exposure, fiber number and mean fiber length of bilateral ACx and right CA1 in NIHL were significantly less than HCs group (Figure 4A–F). The mean fiber length of left CA1 and right CA2 in the NIHL group was much lower than the HCs group (Figure 4H,J). We did not take the CA3 subregion into consideration because of its relatively small volume, and manual drawing led to certain deviations.

### 3.6 Correlation analysis between behavioral tests and basic information

**FIGURE 5:**
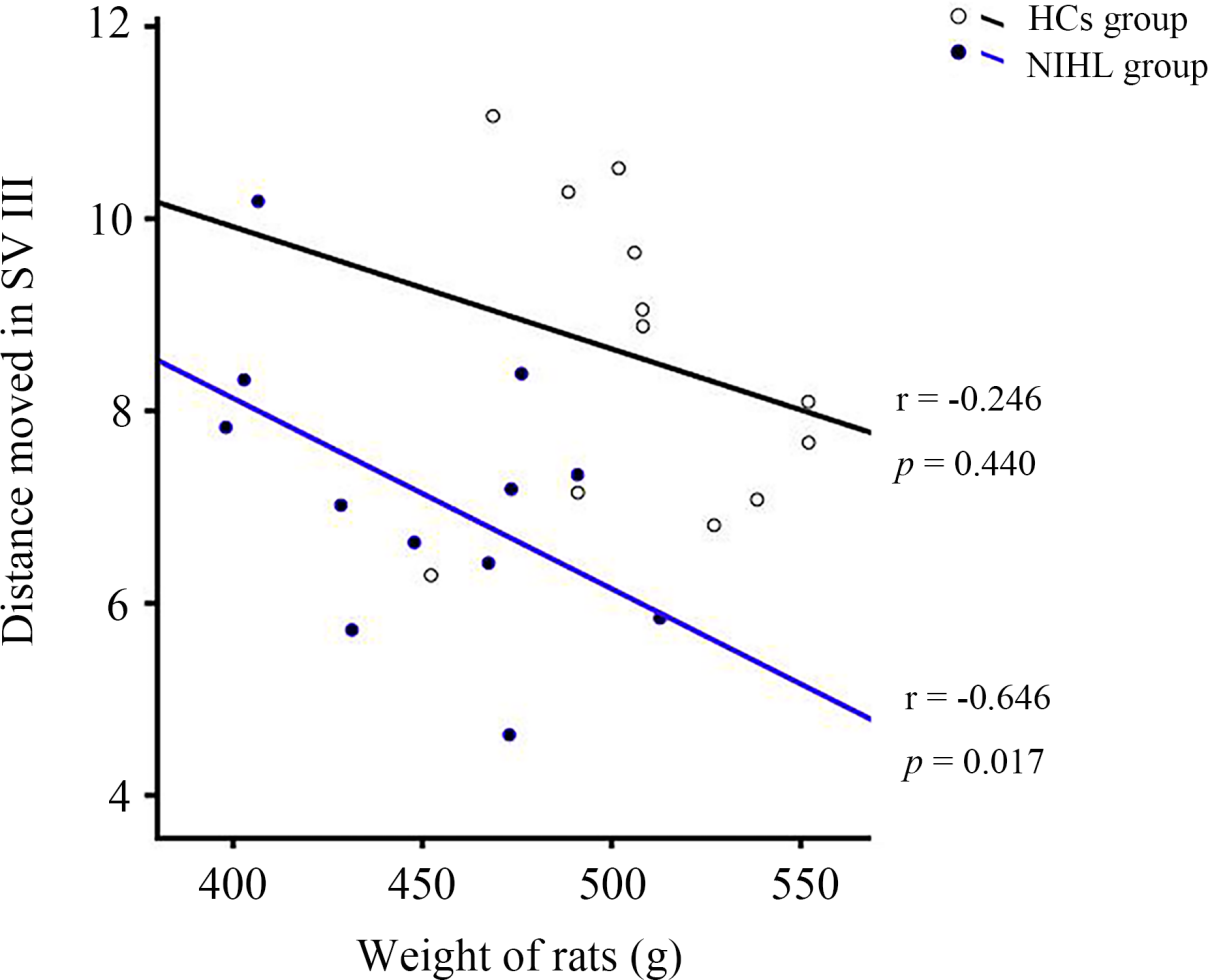
Correlation between body weight and distance moved in SV III. Inserted lines are the results of linear regressions.

A Pearson correlation analysis was performed to verify whether the basic information is correlated with the outcome of MWM tests. A significantly negative correlation was observed between the body weight and swimming distance in SV III in the spatial orientation experiment for each NIHL individual (Figure 5, *r* = −0.646, *p* = 0.017). However, no other correlation relationships were obtained in the present study.

## 4 DISCUSSION

Mapping the borders between sensory‐deprived diseases and neurodegeneration has proved challenging for human research. In the current study, NIHL rats with impaired memory exhibited a distinct network signature, characterized by widely distributed, marked reductions in temporally coherent FC with the bilateral ACx, as well as degraded structural fibers. By comparison, we reported weight loss in NIHL rats and the anticorrelation between body weight and behavioral characteristics. The implications of these findings are elaborated below, including the concept that network disruption associated with cognitive decline reflects a unique neurobiological condition of sensory‐deprived diseases, and the related idea that optimally healthy cognition is enabled by an active neuroadaptive trajectory.

Interestingly, the negative correlation between body weight and distance moved in SV III provided us with a new insight into the relationship between dementia and body weight. Human studies indicated that unintentional body weight loss was common in patients with dementia and is linked to cognitive impairment and poorer disease outcomes,^18^ and it was an independent risk factor for dementia‐related mortality.^19^, ^20^ Moreover, body weight was also found to contribute to predicted changes in hippocampal redox ratio, especially in aging animals.^21^ There is a body of literature demonstrating selective aging‐related vulnerability to oxidative stress in the hippocampus, suggesting that peripheral hearing loss and hippocampal redox changes may share common mechanisms which are highly sensitive to metabolic insults.^22^, ^23^ These data pointed to the possibility of using body weight as a window into the oxidative state of hippocampal neurons. Kim et al.^24^ have evaluated the effects of body mass index on hearing loss in large amounts of adults and found that the rates of hearing were significantly in underweight and obese than in normal‐weight adults. Combining all, the causality link between hearing loss‐induced cognitive impairments and body weight is needed to be further investigated, and weight loss might be a statistical marker of neurodegeneration and associated cognitive decline.

fMRI has clear advantages for auditory characterizations and becomes a vital tool to characterize the auditory pathway in rodents, as it is non‐invasive, provides relatively high spatial resolution, and lends itself to repetitive studies, albeit relying on an indirect neurovascular coupling to deliver its information.^25^, ^26^ Recent findings have suggested that the Tu, in addition to receiving input from the olfactory bulb,^27^ also serves to integrate reward and motivation information from other limbic structures.^28^, ^29^ Taking account of its morphology, chemistry, and anatomical connectivity, the Tu has a trilaminar structure containing distinct layers of neurons and is interconnected with numerous brain regions, including neighboring cognitive and reward‐related limbic structures, such as the nucleus accumbens and ventral pallidum as well as the olfactory bulb, the prefrontal cortex, thalamus, and hippocampus.^30^, ^31^

The limbic system is involved in many emotions and motivations, and the hippocampus is a major limbic region, receiving direct or indirect neural input from the central auditory system,^32^ functioning in long‐term memory.^33^ In an odor‐place‐associated task, neural activity recorded from the entorhinal cortex (EC) and CA1 was thought to depend on the interfacing of the hippocampus with inputs from the olfactory bulb and piriform cortex via the EC.^34^, ^35^ During encoding and retrieval of declarative memories, entorhinal and hippocampal circuits are thought to interact via theta and gamma oscillations,^36^ which in awake rodents predominate frequency spectra in both regions. Since MWM has become the most extensively used protocol to study hippocampus‐dependent spatial learning and memory in rodents,^37^ decreased FC between ACx and hippocampus, Tu may reveal the potential mechanism of NIHL‐induced cognitive impairment. Although previous studies often report that early auditory deprivation or congenital deafness contributes to cross‐modal reorganization in the auditory‐deprived cortex^38^ and aging‐related hearing loss is strongly linked to cognitive impairment,^39^ few studies were concerned about incident dementia induced by post‐lingually hearing loss because of clinical benefits from cochlear prosthetics.

In the present study, we have detected the deficits of microstructural integrity in ACx and HIP using DTI analysis. Mao et al.^40^ established a rat model of hearing loss and tinnitus by exposing to blast noise, then explored alterations to axonal and myelin integrity of auditory brain areas at 2 and 4 weeks following blast exposure. Significant structural changes were found in the IC and medial geniculate body, but not the ACx. Another rat model of conductive hearing loss only found the reduced lateral lemniscus (LL) fiber tract in the duration of 1 week.^11^ These inconsistencies with our findings are probably due to the progression of hearing loss since our research followed up for 6 months and tracked the process of brain remodeling underlying NIHL.

The FC data suggested that hearing loss decreased functional connections in a broad‐neural network that includes core auditory structures extending from LL, through IC to ACx consistent with previous studies implicating these central auditory structures in deafness.^41^, ^42^ The IC of the auditory midbrain integrates the majority of ascending auditory information from lower brainstem regions. It receives prominent long‐range inhibitory input from the LL, a region thought to be important for temporal pattern discrimination.^43^ What is more, the FC between ACx and M1 was disrupted, which may indirectly reflect neural plasticity. Although sensory and motor systems support different functions, both systems can exhibit topographic reorganization of the cortex following training or injury.^44^ Unexpectedly, our data expanded that PFL is involved in regulating hearing loss. The cerebellum is mainly involved in motor planning and control, and some cerebellar regions such as the PFL and vermis receive inputs from auditory centers.^45^

Our study also has some limitations. Firstly, we did not find any correlations between MRI and behavioral tests, which may be due to the limited sample size. And we applied uncorrected *p‐*value in imaging analysis, as it is reported that a small sample size might contribute to a low signal‐to‐noise ratio and poor statistical power.^11^ Future studies should expand it and address data quality. Secondly, although MRI techniques have been widely used in neuropsychiatric disorders, a combination of histopathological techniques is also needed in further experiments.

## 5 CONCLUSION

Our finding proposed an auditory–limbic–cerebellum model to elucidate the neural patterns of NIHL‐associated cognitive impairments. Combining functional and structural MRI might provide different aspects of brain plasticity, which could serve a potential role in non‐invasive diagnosis and therapy evaluation of NIHL in the future.

## AUTHOR CONTRIBUTIONS

Xiao‐Min Xu collected data and wrote this manuscript; Yuan Feng helped with data collection; Jian Wang and Richard Salvi provided scientific advice; Xindao Yin helped with discussion and revision; Yu‐Chen Chen and Jun Gao contributed equally and participated in writing or technical editing of the manuscript.

## FUNDING INFORMATION

This work was supported in part by the Doctoral Program of Entrepreneurship and Innovation in Jiangsu Province (JSSCBS20211544), Xinghuo Talent Program of Nanjing First Hospital, and Nanjing Special Fund for Health Science and Technology Development (No. YKK21133).

## CONFLICT OF INTEREST

The authors declare that there is no potential conflict of interest regarding the publication of this study.

## Supporting information


Tables S1‐S2

## Data Availability

Data will be made available upon request.
