# Supplementary material for: Auditory–limbic–cerebellum interactions and cognitive impairments in noise‐induced hearing loss
Source: CNS Neurosci Ther. 2022 Nov 15;29(3):932–40. doi: 10.1111/cns.14028 (PMC9928548; doi:10.1111/cns.14028)
Supplement: Supplementary file 1 — Tables S1‐S2 [file CNS-29-932-s001.docx]

Table S1. Auditory thresholds of NIHL group and HCs group at frequencies of 2,4,8,16, 32 kHz.

| **ABR test** | **NIHL group (dB SPL)** | | **HCs group (dB SPL)** | | ***p* value** |
| --- | --- | --- | --- | --- | --- |
| 2 kHz | | 87.31 ± 3.88 | | 43.33 ± 7.18 | <0.001 |
| 4kHz | | 89.62 ± 1.39 | | 30.00 ± 4.26 | <0.001 |
| 8kHz | | 89.62 ± 1.39 | | 16.25 ± 3.77 | <0.001 |
| 16kHz | | 89.23 ± 1.88 | | 19.58 ± 5.82 | <0.001 |
| 32kHz | | 88.46 ± 2.40 | | 31.67 ± 7.49 | <0.001 |

NIHL, noise induced hearing loss; HCs, healthy controls; ABR, auditory brainstem response.

Table S2. Significant brain regions of functional connections using bilateral primary auditory cortex as the seed at 6 months post noise exposure.

| **Seed region** | **Brain region** | **Left brain** | | **Right brain** | |
| --- | --- | --- | --- | --- | --- |
|  |  | **Cluster size** | **t value** | **Cluster size** | **t value** |
| **Covariance (body weight)** | | | | | |
| ACx | PFL | / | / | 36 | -5.1996 |
|  | IC | 11 | -4.6386 | / | / |
|  | LL | / | / | 14 | -4.7311 |
|  | HIP | / | / | 17 | -4.9054 |
|  | M1 | / | / | 12 | -5.6358 |
|  | Tu | 24 | -5.6183 | / | / |
| **No covariance (body weight)** | | | | | |
| ACx | PFL | / | / | 10 | -4.643 |
|  | HIP | / | / | 30 | -5.2901 |
|  | AIP | 10 | -4.5887 | / | / |

ACx, auditory cortex; PFL, parafloccular lobe of cerebellum; IC, inferior colliculus; LL, lateral lemniscus; HIP, hippocampus; M1, primary motor cortex; Tu, olfactory tubercle; AIP, agranular insular cortex. *P*<0.001, uncorrected.
